# Differentiation and Distribution of Cordyline Viruses 1–4 in Hawaiian ti Plants (*Cordyline fruticosa* L.)

**DOI:** 10.3390/v5071655

**Published:** 2013-07-05

**Authors:** Michael Melzer, Caleb Ayin, Jari Sugano, Janice Uchida, Michael Kawate, Wayne Borth, John Hu

**Affiliations:** 1 Department of Plant and Environmental Protection Sciences, University of Hawaii, 3190 Maile Way, Honolulu, HI 96822, USA; E-Mails: cayin@hawaii.edu (C.A); johnhu@hawaii.edu (J.U.); mike@hpirs.stjohn.hawaii.edu (M.K.); borth@hawaii.edu (W.B.); juchida@hawaii.edu (J.H.); 2 Department of Plant and Environmental Protection Sciences, University of Hawaii, 45-260 Waikalua Road, Kaneohe, HI 96744, USA; E-Mail: suganoj@ctahr.hawaii.edu

**Keywords:** cordyline virus, velarivirus, ti ringspot, herbarium sampling

## Abstract

Common green ti plants (*Cordyline fruticosa* L.) in Hawaii can be infected by four recently characterized closteroviruses that are tentative members of the proposed genus *Velarivirus*. In this study, a reverse-transcription polymerase chain reaction (RT-PCR) assay developed to detect and distinguish Cordyline virus 1 (CoV-1), CoV-2, CoV-3, and CoV-4 was used to determine: (i) the distribution of these viruses in Hawaii; and (ii) if they are involved in the etiology of ti ringspot disease. One hundred and thirty-seven common green ti plants with and without ti ringspot symptoms were sampled from 43 sites on five of the Hawaiian Islands and underwent the RT-PCR assay. Eleven ornamental ti varieties were also sampled and assayed. Based on this survey, it appears none of the CoVs are involved in the etiology of ti ringspot. The observation of a non-uniform geographic distribution of the CoVs in common green ti, combined with the presence of CoVs in seed-derived ornamental varieties, suggests active vector transmission. Eight herbarium specimens collected between 1903 and 2003 from plants on the island of Oahu also underwent the RT-PCR assay. Amplifiable RNA was isolated from accessions collected in 1985 or later, however only the 2003 accession was found to harbor CoVs.

## 1. Introduction

The family *Closteroviridae* is a group of related plant viruses with long, flexuous virions encapsidating a large, positive-sense genomic RNA. The family is currently divided into three genera [1,2]. Members of the genera *Closterovirus* and *Ampelovirus* have monopartite genomes and are transmitted by aphids and mealybugs, respectively. Members of the genus *Crinivirus* have multipartite genomes and are transmitted by whiteflies. Little cherry virus 1 (LChV-1), Grapevine leafroll-associated virus 7 (GLRaV-7), and Cordyline virus 1 (CoV-1) are monopartite closteroviruses that form a distinct clade within the family [3]. This has led to proposals for the formation of a fourth genus, with the proposed name *Velarivirus* [3,4]. The recently characterized CoV-2, CoV-3, and CoV-4 would also join this proposed genus [5]. Unlike other genera in the family, no insect vector has been associated with any of the putative members of this proposed genus.

In Hawaii, the common green variety of ti (*Cordyline fruticosa* L.) was introduced by early Polynesian settlers and is a popular ornamental in residential settings that has also become naturalized in Hawaii’s forests. Vegetatively-propagated due to sterility [6], it is also the most prominent variety grown commercially. Other more recently introduced ti varieties that have various leaf colors and shapes and that are capable of sexual reproduction are also grown commercially for landscaping and flower arrangements. 

First observed in 2009, ti ringspot is an emerging disease mainly affecting the common green variety of ti in Hawaii, but occasionally ornamental varieties as well. The causal agent of this disease has not yet been identified, however, CoV-1, CoV-2, CoV-3, and CoV-4 have been shown to infect Hawaiian ti [5,7]. A preliminary survey suggested that CoV-1 is not involved in ti ringspot [7], however a more robust survey must be conducted to exclude CoV-1 as the causal agent as well as to determine if CoV-2, CoV-3, or CoV-4 are associated with ti ringspot. In this study, a reverse-transcription polymerase chain reaction (RT-PCR) assay was developed to detect and distinguish the CoVs. Common green ti plants with and without ti ringspot symptoms were sampled from across the Hawaiian islands and assayed for the presence of these viruses. In addition, ornamental ti varieties and tissue from herbarium specimens were assayed for the presence of CoVs.

## 2. Results and Discussion

### 2.1. Differentiation of CoVs

**Figure 1:**
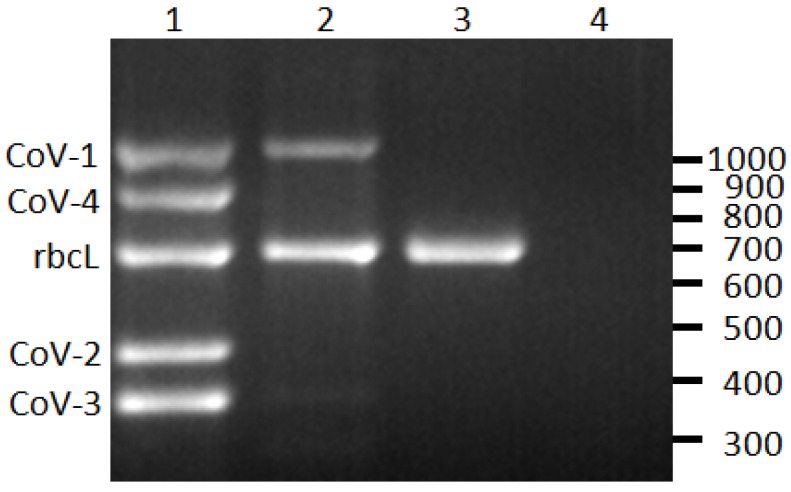
Reverse-transcription PCR detection of Cordyline viruses (CoVs) 1–4 infecting *C. fruticosa*. Lane 1, plant infected with all four CoVs; lane 2, plant infected with CoV-1; lane 3, CoV-free plant; lane 4, non-template control. The rubsico large subunit (rbcL) is an internal positive control for the assay. The scale on right indicates size in base pairs.

Using the sequence data from the Heat Shock Protein 70 homolog (HSP70h) gene region of CoV-1, CoV-2, CoV-3, and CoV-4, virus-specific RT-PCR primer sets were designed (Table 1). These primers were validated by sequencing select amplification products which were found to be >97% identical to the intended target sequence (data not shown). To serve as a positive internal control, primer set 1220/1221 was designed to target the plastid ribulose-1,5-bisphosphate carboxylase large subunit gene (*rbcL*) of *C. fruticosa* (Figure 1). Amplification products of the correct size were observed from all plant samples unless otherwise noted.

**Table 1:** Primers used for the detection and differentiation of Cordyline viruses (CoVs) infecting ti plants (*Cordyline fruticosa*) plants in Hawaii.

Target	Primer	5' to 3' Sequence (polarity)	Amplicon (bp)
CoV-1	1220	CCTTCTTCGATAATAAGGTGGTG (+)	1,075
	1221	AGGTGTAAGGAATTGGCATTGG (−)	
CoV-2	1216	GATTGTCAAATGGGAGGAATTGG (+)	465
	1217	AGATACGGGAGAGATAAAGTTGG (−)	
CoV-3	1214	CATATGTCTGGTATTGGTACGG (+)	375
	1215	CCAAGGAAAAGATCACCTTCTG (−)	
CoV-4	1218	TACGATCTAAAAAGATGGGTCGG (+)	894
	1219	GGATCTTCTAGAAGAATGTGGAG (−)	
*C. fruticosa* (*rbcL*)	1212	AGTATGGTCGTCCTTTTTTGGG (+)	700
	1213	CATCTCCAAAGATTTCGGTTAGG (−)	
Closterovirus *HSP70h*	249	GANVHRTCRAAMGTSCCTCCNCCRAARTC (−)	~575
	250	GGARGTNGGNWWHGAMTTYGGNACNAC (+)	
	1321	GAYCTNAARMGNTGGGTNGGNG (+)	~410

### 2.2. CoVs and ti Ringspot Disease

**Figure 2:**
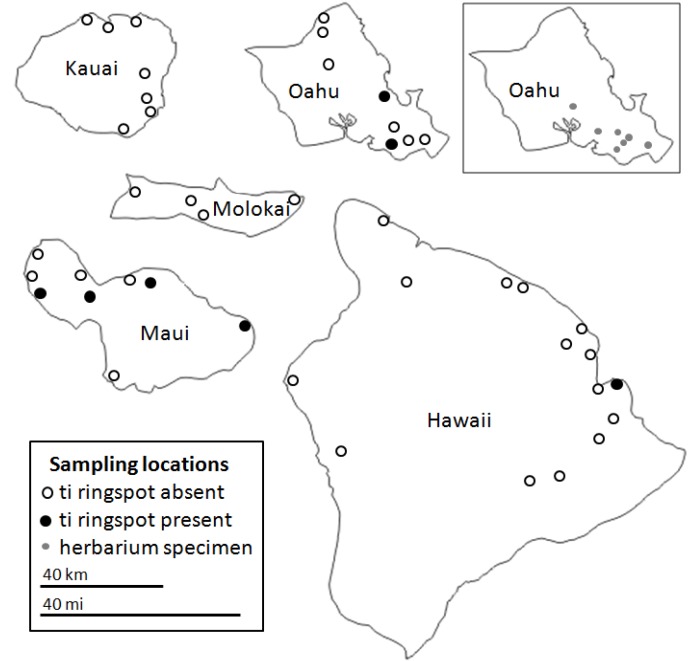
Locations of where *C. fruticosa* plants were sampled on five of the major Hawaiian Islands. The location of where some herbarium specimens were collected on Oahu (inset) is approximate due to a lack of detailed information. Relative sizes of the islands are to scale, but their geographic locations relative to each other are not accurate.

A total of 137 common green ti plants from 43 sites on the islands of Kauai, Oahu, Molokai, Maui, and Hawaii were assessed for the presence of the four CoVs using the RT-PCR assay (Figure 2, Table 2). Of these plants, 22 displayed symptoms of ti ringspot whereas the remaining 115 plants were without ringspot symptoms. There was no clear relationship between the presence of any CoVs, or combination thereof, and ti ringspot symptoms. For example, 5 symptomatic plants were found to be CoV-free. Conversely, 2 asymptomatic plants harbored all four CoVs (Table 2). This supports our previous observations indicating that CoV-1 was not directly involved in the etiology of ti ringspot disease [7]. This result is not surprising since members of the family *Closteroviridae* are phloem-associated viruses that damage the vasculature of their host, causing chlorosis, stunting, and wilt/decline [1], but typically do not induce ringspot symptoms. 

**Table 2:** Cordyline virus (CoV) status of common green ti, *Cordyline fruticosa*, plants with or without ringspot symptoms in the Hawaiian Islands.

Virus	Symptomatic						Asymptomatic					
	Kauai	Oahu	Molokai	Maui	Hawaii	Total	Kauai	Oahu	Molokai	Maui	Hawaii	Total
Negative	-	1	-	3	1	5	4	6	8	8	20	46
CoV-1	-	-	-	5	-	5	-	12	1	-	4	17
CoV-2	-	-	-	1	-	1	3	1	-	5	1	10
CoV-3	-	-	-	-	-	-	10	-	-	1	3	14
CoV-4	-	-	-	-	-	-	-	1	1	-	1	3
CoV-1 + CoV-2	-	-	-	-	-	-	-	1	-	-	3	4
CoV-1 + CoV-3	-	-	-	-	-	-	-	-	-	-	1	1
CoV-1 + CoV-4	-	-	-	3	-	3	-	1	1	-	1	3
CoV-2 + CoV-3	-	-	-	-	-	-	3	1	-	-	-	4
CoV-2 + CoV-4	-	1	-	-	-	1	-	-	-	-	3	3
CoV-3 + CoV-4	-	-	-	-	-	-	1	-	-	-	-	1
CoV-1 + CoV-2 + CoV-3	-	-	-	-	-	-	-	-	-	-	2	2
CoV-1 + CoV-2 + CoV-4	-	1	-	1	-	2	-	2	2	-	-	4
CoV-1 + CoV-3 + CoV-4	-	3	-	-	-	3	-	-	-	-	-	-
CoV-2 + CoV-3 + CoV-4	-	-	-	-	-	-	-	-	-	-	1	1
CoV-1 + CoV-2 + CoV-3 + CoV-4	-	2	-	-	-	2	-	-	-	1	1	2
Total	-	8	-	13	1	22	21	25	13	15	41	115

### 2.3. Spatial and Varietal Distribution of CoVs

As single or mixed infections, the incidences of CoV-1, CoV-2, CoV-3, and CoV-4 in common green ti samples were 36%, 26%, 22%, and 20%, respectively. Green ti plants on the island of Kauai had the highest incidence of CoVs (81%), followed by the islands of Oahu (79%), Maui (61%), Hawaii (50%), and Molokai (38%). The statewide incidence of CoVs in common green ti was 63% (86/137). In a preliminary survey of CoV-1, we demonstrated that this virus is widespread on Oahu and Maui, and speculated that it may have been present in the initial propagative materials brought to Hawaii by early Polynesian settlers [7]. The expanded survey conducted for this study supports the observation that CoV-1 is widespread on Oahu and Maui, but does not support the hypothesis that CoV-1, or any of the closteroviruses, were present in the ti materials brought to Hawaii by early Polynesian settlers. Almost 37% of the common green ti plants tested were CoV-free. Given the scarcity of common green ti plants derived from seed in Hawaii [6] which would putatively represent closterovirus-free germplasm, it is most probable that Hawaii’s ti plants were initially closterovirus-free and are now being infected, likely by a viruliferous insect vector.

Eleven ornamental ti plants, each representing a different variety originally derived from seeds, were also evaluated for the presence of CoVs (Table 3). None of these plants displayed ringspot symptoms. Eight of these plants were found to be infected with at least one of the closteroviruses. CoV-4 was the most prevalent of the closteroviruses in ornamental ti with an incidence of 73%, followed by CoV-1 and CoV-2 (18%), and CoV-3 (9%). The presence of the CoVs in seed-derived germplasm further supports our contention that these viruses are being actively spread by a viruliferous vector. 

**Table 3:** Detection of Cordyline viruses (CoVs) in ornamental *Cordyline fruticosa* varieties.

Variety	CoV-1	CoV-2	CoV-3	CoV-4
Apple Juno	+	−	−	+
Charles Nii #3	−	−	−	−
Haole Girl	−	−	−	+
Harold Yamamoto #12	−	−	+	+
Heather Spray	−	−	−	−
Kahili	+	−	−	+
Kalani Akaka	−	−	−	+
Kauai Beauty (miniature)	−	+	−	+
Lilian Olivera	−	−	−	+
Mauna Kea (miniature)	−	−	−	−
Tachibana	−	+	−	+
Total	2/11 (18%)	2/11 (18%)	1/11 (9%)	8/11 (73%)

### 2.4. Herbarium Specimens

Specimens of *C. fruticosa* collected between 1903 and 2003 on the island of Oahu and stored at the Bishop Museum’s Herbarium Pacificum were evaluated for the presence of CoVs using the RT-PCR assay (Table 4). Fragmented plant material stored in packets alongside the specimen was the only tissue available for destructive sampling. This tissue was mostly composed of detached flowers as opposed to petiole tissue used in the spatial and varietal surveys described above. To determine whether CoVs could be detected in flower tissue, the single inflorescence of a common green ti plant previously shown to be infected with CoV-1 and CoV-4 using leaf petiole tissue underwent the RT-PCR assay. Both CoV-1 and CoV-4, as well as *rbcL* RNA could be detected in the inflorescence (data not shown), demonstrating that flower tissue from herbarium specimens may also be suitable for testing. The *rbcL* RNA template was successfully amplified from the two most recently collected specimens (2003 and 1985), but not from any specimens collected in 1970 or earlier. CoV-3 and CoV-4 were detected in the 2003 specimen, but no CoVs were detected in any of the other specimens. Although DNA is commonly isolated from herbarium and other preserved tissues (reviewed in [8,9]), there are few studies reporting the isolation of RNA from such tissue, presumably due to its relative instability compared to DNA. Malmstrom *et al.* [10], however, were able to detect Barley yellow dwarf virus from Avena fatua herbarium specimens collected as early as 1917. Similarly, Guy [11] was able to detect peach latent mosaic viroid (PLMVd) and *rbcL* sequences from peach (*Prunus persica*) samples collected in 1956. The inability to detect either *rbcL* or CoV RNA in samples collected in 1970 or earlier indicates that some degree of RNA degradation has occurred in these older samples. The rate of RNA degradation may be plant host-dependent as Guy [11] was able to detect *rbcL* sequences in *P. persica* specimens but not from another *Prunus* sp. specimen or from an apple specimen collected at the same time and archived on the same herbarium sheet. The CoV and *rbcL* amplification products in this study ranged from 375 to 1,075 bp in length. The targeting of shorter amplification products may increase the chances of amplification, particularly if the RNA molecules are only partially degraded. 

**Table 4:** Detection of Cordyline viruses (CoVs) and the rubisco large subunit gene (*rbcL*) in herbarium specimens of *Cordyline fruticosa* archived at the Bishop Museum’s Herbarium Pacificum.

Specimen ID	Date Collected	Tissue (weight)	Location	*rbcL*	CoV-1	CoV-2	CoV-3	CoV-4
BISH 697966	Jan 13, 2003	leaf petiole (15 mg)	Lyon Arboretum	+	−	−	+	+
BISH 504184	Jan 19, 1985	flower, leaf (9 mg)	Manoa Falls	+	−	−	−	−
BISH 504185	Jan 19, 1985	flower (7 mg)	Manoa Falls	−	−	−	−	−
BISH 76555	Dec 22, 1970	flower (5 mg)	Lyon Arboretum	−	−	−	−	−
BISH 487514	Apr 9, 1962	flower, leaf (17 mg)	University of Hawaii Manoa Campus	−	−	−	−	−
BISH 06727	Dec 10, 1945	flower, pedicel (27 mg)	University of Hawaii Manoa Campus	−	−	−	−	−
BISH 446936	Mar 22, 1936	flower (11 mg)	Kipapa Gulch	−	−	−	−	−
BISH 121224	Jan 12, 1920	flower (5 mg)	Wailupe Valley	−	−	−	−	−
BISH 92012	Dec 29, 1909	flower (8 mg)	Hillebrands Glen (Nuuanu Valley)	−	−	−	−	−
BISH 121219	Dec 13, 1903	flower (6 mg)	Moanalua Valley	−	−	−	−	−

### 2.5. Degenerate Primer RT-PCR

Eighteen of the common green ti and the three ornamental ti samples that tested negative using the CoV-specific assays, as well as two samples which each tested positive for all four CoVs, were subjected to RT-PCR analysis using degenerate primer sets 249/250 [12] and 249/1321. No products were generated for any of the 21 negative samples with either primer set. In contrast, prominent RT-PCR products were generated for CoV-infected samples with both primer sets (data not shown). The 249/1321 products for these two samples were cloned and 12 clones for each product were sequenced. All clones were >86% identical to the *HSP70h* sequence of one of the four described CoVs. Taken together, this suggests that if additional CoVs exist, they are either rare, part of a mixed infection, or are not present in Hawaii’s ti plants.

## 3. Experimental

### 3.1. Differentiation of CoVs

An RT-PCR assay was developed to detect and differentiate the closteroviruses infecting common green ti plants in Hawaii. Primer sets targeting CoV-1 (primers 1220/1221), CoV-2 (1216/1217), CoV-3 (1214/1215), and CoV-4 (1218/1219) were designed (Table 1). Primer set 1212/1213 targets *rbcL* of *C. fruticosa* and serves as a positive control for the assay (Table 1). Total RNA was isolated from 100 mg of leaf petiole tissue using a NucleoSpin^®^ RNA Plant kit (Macherey-Nagel, Bethlehem, PA, USA) following the manufacturer’s directions. RNA (2 µL, representing 4 mg of leaf tissue) was incubated at 72 °C for 10 min with 50 ng of random hexamers and quickly chilled on ice. To create complementary DNA (cDNA), a 20 μL reaction was set up containing the RNA, random hexamers, 50 mM Tris-HCl (pH 8.3), 75 mM KCl, 3 mM MgCl_2_, 10 mM DTT, 0.2 mM of each dNTP, 20 units of RNasin^®^ ribonuclease inhibitor (Promega, Madison, WI, USA), and 200 units of M-MLV reverse transcriptase (Promega). After a 5 min incubation at room temperature, the reaction was incubated at 42 °C for 60 min. Hot-start PCR was performed using 1 μL of cDNA from this reaction as template, 5 pmol of each primer (Table 1), and 2X Immomix^®^ (Bioline, Taunton, MA, USA). PCR conditions were 95 °C for 7 min; 40 cycles of 95 °C for 60 s, 53 °C for 60 s, 72 °C for 60 s; and 72 °C for 7 min. The five PCR reactions for each RNA sample were combined and resolved by electrophoresis in a 1.5% to 2% (w/v) agarose gel. Selected products were cloned using the pGEM^®^-T Easy vector (Promega) and sequenced at the University of Hawaii’s Advanced Studies in Genomics, Proteomics, and Bioinformatics laboratory.

### 3.2. Geographical and Varietal Distribution

A total of 137 common green ti plants with or without ringspot symptoms were sampled from 43 sites on the islands of Kauai, Oahu, Molokai, Maui, and Hawaii. Between one and five plants were sampled at each location. Large, naturalized plants in remote or forested areas were preferentially sampled over smaller ornamental plants growing in residential or urban areas that could possibly have been obtained from a nursery. Samples were kept on ice until brought to the laboratory where they were stored at −20 °C. Eleven ornamental varieties of ti growing at the Lyon Arboretum on the island of Oahu were also sampled. No ringspot symptoms were observed on any of the ornamental ti varieties sampled in this study.

### 3.3. Herbarium Specimens

To determine if CoVs could be detected in preserved plant tissues, ten ti accessions archived at the Bishop Museum’s Herbarium Pacificum were sampled under the guidance of the collection manager. Between 5 and 27 mg of tissue from ti accessions collected on the island of Oahu between 1903 and 2003 underwent RNA isolation using TRIzol^®^ Reagent (Life Technologies Corp., Grand Island, NY, USA) following the manufacturer’s instructions. Nucleic acids were re-suspended in 50 μL of nuclease-free water and RT-PCR was performed as described above, except that 5.5 μL of RNA was used for cDNA synthesis.

### 3.4. Degenerate Primer RT-PCR

To determine if any additional closteroviruses are present in ti plants, degenerate primer PCR assays were performed on the cDNA of selected total RNA samples from above. The negative-sense primer 249, which targets motif C of closteroviral *HSP70h* was paired with the positive-sense primers 250 or 1321 which target motif A and a conserved region between motifs A and B of closteroviral *HSP70h*, respectively (Table 1) [12]. PCR conditions were 95 °C for 7 min; 40 cycles of 95 °C for 60 s, 42 °C for 60 s, 72 °C for 60 s; and 72 °C for 7 min. Products were resolved by electrophoresis in a 1% (w/v) agarose gel. 

## 4. Conclusions

The results of this study provide evidence that CoV-1, CoV-2, CoV-3, and CoV-4 are not directly involved in the etiology of ti ringspot disease. Although these viruses are widespread in Hawaii, it appears that they were introduced after ti plants were established in Hawaii, and that they are being actively transmitted, likely by an insect vector. As such, these viruses would represent the only members of the proposed genus *Velarivirus* with evidence of natural transmission. This study represents one of the few examples of RNA virus detection in herbarium specimens using molecular techniques. It is clear that some CoVs have been present in Hawaii for at least 10 years. 
